# Exploring Pre-Service Mathematics Teachers’ Diagnostic Thinking During AI-Supported Task Design: A Configurational Mixed-Methods Pilot Study

**DOI:** 10.3390/jintelligence14090227

**Published:** 2026-09-20

**Authors:** Chunxia Qi, Jingyu Lin, Leyao Wen, Qi Huang

**Affiliations:** 1 Faculty of Education, Beijing Normal University, Beijing 100875, China; jingyu@mail.bnu.edu.cn (J.L.); mutila2021@163.com (L.W.); 2 College of Education, Purdue University, West Lafayette, IN 47907, USA; huan2304@purdue.edu

**Keywords:** pre-service mathematics teachers, diagnostic thinking, human–AI collaboration, mathematical task design, fuzzy-set Qualitative Comparative Analysis, teacher education

## Abstract

Developing pre-service mathematics teachers’ ability to understand students’ mathematical thinking and anticipate students’ difficulties remains a challenge in teacher education. This exploratory mixed-methods study involved 21 pre-service mathematics teachers in a six-week AI-supported mathematical task design module. The study combined pre–post assessment, fuzzy-set Qualitative Comparative Analysis (fsQCA), and qualitative analysis of participants’ AI interactions and reflections. The key findings were as follows: (1) Participants showed significantly higher post-test scores in overall diagnostic thinking and in perception, interpretation, and judgement than at pre-test. (2) The configurational analysis identified a comparatively stable pattern associated with high post-intervention diagnostic thinking, combining lower initial diagnostic thinking with high AI acceptance and high prompting knowledge density. (3) Qualitative evidence further showed that AI served as an interactive resource for exploring mathematical and pedagogical considerations, evaluating generated suggestions, and revising task designs. Exploratory comparisons suggested that AI use could range from broader knowledge-oriented support to more selective verification and refinement. The findings highlight the heterogeneous nature of AI-supported professional learning and underscore the importance of critical evaluation and professional agency in teacher–AI collaboration.

## 1. Introduction

The ability to understand students’ mathematical thinking and to design tasks that are appropriately aligned with their mathematical knowledge and learning needs is an important aspect of mathematics teachers’ professional competence (Brunner et al., 2024; Ministry of Education of China, 2022; OECD, 2019). Diagnostic thinking, as a situation-specific process of perceiving, interpreting, and judging information relevant to learners and instructional tasks, enables teachers to assess task demands, anticipate potential student difficulties, and make informed judgments about students’ possible mathematical understanding (Brunner et al., 2021; Herppich et al., 2018; Südkamp et al., 2012). For pre-service mathematics teachers (PSTs), the development of this competence is particularly important because they must learn to connect mathematical knowledge with knowledge of how students may understand, interpret, and struggle with mathematical ideas. In the context of China, where high-stakes examinations exert a strong influence on teaching and learning practices (Qi et al., 2022), the ability to design mathematically meaningful and appropriately challenging tasks is especially relevant to future teachers’ professional preparation.

However, developing diagnostic thinking is challenging for PSTs. Although prior research has examined the nature and measurement of teachers’ diagnostic thinking (Brunner et al., 2021; Herppich et al., 2018; Südkamp et al., 2012), PSTs often have limited opportunities to repeatedly anticipate students’ possible mathematical strategies, difficulties, and misconceptions before entering the teaching profession (Levin et al., 2009; Xu & Brown, 2016). Moreover, the professional knowledge that teachers mobilize when making such judgments is often difficult to observe directly. Consequently, less is known about how structured learning activities can support the development of PSTs’ diagnostic thinking and how learners with different initial levels of this competence may develop through the same learning experience.

Recent advances in AI, particularly Large Language Models (LLMs), create new opportunities for PSTs to engage in repeated, low-risk task design activities, explore alternatives, and receive immediate responses (Frieder et al., 2023; Holmes & Miao, 2023). However, the educational value of AI output is not automatic. Because LLMs’ responses are shaped by users’ prompts, their relevance depends on how PSTs articulate mathematical and pedagogical considerations, refine their requests, and critically evaluate the results. AI-supported mathematical task design should therefore be understood as an iterative human–AI process. Through cycles of prompting, evaluation, reflection, and revision, PSTs can coordinate mathematical content, curriculum expectations, task difficulty, students’ prior knowledge, possible strategies, and misconceptions while making their professional reasoning more explicit and reconsidering how students may respond to a task.

Nevertheless, the benefits of AI-supported learning may vary across learners and may depend on combinations of learner characteristics and interactional conditions. Prior research suggests that learners with different levels of prior knowledge may benefit from different forms of scaffolding (Chernikova et al., 2020), while technology acceptance may also shape engagement with AI-supported learning (Nikolopoulou et al., 2020; Tan et al., 2025). However, little is known about how different combinations of these conditions are associated with high post-intervention diagnostic thinking. A configurational perspective is therefore needed to examine how these conditions are configured in relation to successful professional learning.

A further challenge concerns the interactional processes through which such development occurs. Existing research on AI in teacher education has increasingly examined AI acceptance, usage, and prompting behavior (Engelbrecht & Borba, 2024; Lo, 2023; Tan et al., 2025; Wardat et al., 2023). However, less is known about how teachers with different levels of initial competence engage with AI along different developmental pathways. Variable-oriented approaches may identify the independent associations of individual factors but are less suited to examining the configurational nature of AI-supported professional learning, in which different combinations of learner characteristics and interactional behaviors may be associated with similar outcomes. In addition, quantitative analyses alone provide limited insight into how teachers actually activate and integrate professional knowledge during human–AI interaction.

To address these gaps, this exploratory pilot study examined changes in PSTs’ diagnostic thinking during a six-week AI-supported mathematical task design module, the configurations associated with high post-intervention diagnostic thinking, and the interactional processes through which PSTs engaged with, evaluated, and revised AI-generated suggestions. The research is guided by the following questions:

RQ1: What within-participant changes in diagnostic thinking scores were observed during the AI-supported task design program?

RQ2: What configurations of initial diagnostic thinking, AI acceptance, and prompting knowledge density are associated with high post-intervention diagnostic thinking?

RQ3: How did PSTs engage with, evaluate, and revise AI-generated suggestions during mathematical task design?

## 2. Literature Review

### 2.1. Diagnostic Thinking and Understanding Students’ Mathematical Thinking

#### 2.1.1. Conceptualizing Diagnostic Thinking in Mathematics Education

Diagnostic thinking is an important component of teachers’ professional competence. In educational research, it has generally been conceptualized as a situation-specific cognitive process through which teachers use available information to form judgments about students’ knowledge, performance, and learning needs (Ready & Wright, 2011; Blömeke et al., 2015). Early research on teachers’ diagnostic judgments primarily focused on their ability to accurately estimate students’ expected or actual academic performance (Schrader, 2009; Südkamp et al., 2012). Teachers may also need to judge the difficulty of instructional tasks and anticipate how students are likely to perform on those tasks (Ostermann et al., 2018). From this perspective, diagnostic thinking involves making informed judgments about learners on the basis of available evidence rather than relying solely on general impressions or intuition.

In mathematics education, however, diagnostic thinking involves more than judging whether students are likely to succeed on a task. Teachers also need to understand how students may interpret a mathematical problem, which strategies they may use, what errors they may make, and what these responses may reveal about their underlying mathematical understanding (Chapman, 2014; Hardy et al., 2019; Kron et al., 2021; Leuders et al., 2018). Mathematical thinking is not directly observable. Teachers must therefore infer students’ understanding from observable evidence, such as written solutions, solution strategies, explanations, errors, and other forms of mathematical activity. The central challenge of diagnostic thinking in mathematics education is thus to move from observable student responses to plausible interpretations of the mathematical thinking underlying those responses.

The cognitive processes involved in diagnostic thinking have been examined through the DiaCoM framework proposed by Loibl et al. (2020), which conceptualizes teachers’ diagnostic judgments as involving perception, interpretation, and judgment. Perception refers to attending to and noticing potentially relevant information. In a mathematics classroom, this may involve noticing a particular strategy, error, representation, or explanation in a student’s work. Interpretation involves making sense of the noticed information and relating it to relevant knowledge. Teachers may, for example, consider whether a particular error reflects a computational mistake, a misunderstanding of a mathematical concept, or the application of an alternative but potentially productive strategy (Brunner et al., 2021; Loibl et al., 2020; Rieu et al., 2022). Judgment refers to the formation of a more conclusive evaluation based on the available evidence, such as a judgment about a student’s mathematical understanding or learning needs.

This process is closely related to the literature on teachers’ professional noticing of students’ mathematical thinking. Jacobs et al. (2010) conceptualized professional noticing as involving three interrelated practices: attending to children’s strategies, interpreting their mathematical understandings, and deciding how to respond on the basis of those understandings. Subsequent research has shown that teachers, particularly pre-service teachers, may be able to identify whether a student’s answer is correct or incorrect while experiencing greater difficulty in interpreting the mathematical reasoning underlying the response (Amador et al., 2016). Thus, noticing a student response and understanding the mathematical thinking that corresponded to it represent related but distinct aspects of professional competence.

Although diagnostic thinking and professional noticing have been conceptualized from somewhat different perspectives, both highlight the importance of attending to and interpreting evidence of students’ thinking (Loibl et al., 2020; Jacobs et al., 2010). In this study, this perspective is used to examine how pre-service mathematics teachers understand possible student strategies, errors, and underlying mathematical understandings.

#### 2.1.2. Knowledge and Professional Learning for Developing Diagnostic Thinking

Previous research has linked teachers’ diagnostic thinking to their professional knowledge, particularly their ability to mobilize mathematical and pedagogical knowledge when interpreting students’ mathematical activity (Loewenberg Ball et al., 2008; Baumert et al., 2010; Ostermann et al., 2018). This relationship is particularly evident in research on mathematical knowledge for teaching (MKT). The MKT framework emphasizes that teachers require knowledge that extends beyond the ability to solve mathematical problems themselves (Loewenberg Ball et al., 2008). In particular, knowledge of content and students (KCS) concerns how students are likely to understand particular mathematical ideas, the strategies they may use, and the errors or misconceptions they may develop. Such knowledge is directly relevant to diagnostic thinking because teachers need to connect observable student responses with possible underlying mathematical understandings.

Empirical research has also demonstrated the relationship between teachers’ professional knowledge and their diagnostic judgments. Teachers with stronger professional knowledge may be better able to identify relevant evidence and interpret students’ responses in relation to the mathematical content involved (Baumert et al., 2010; Ostermann et al., 2018). Nevertheless, possessing relevant knowledge does not automatically ensure effective diagnostic thinking. Teachers must also recognize which aspects of a student’s response are informative, determine how these aspects relate to possible mathematical understandings, and evaluate alternative interpretations. Diagnostic thinking therefore involves not only the possession of professional knowledge but also its situated application.

The development of diagnostic thinking has consequently become an important focus of teacher education. Research suggests that diagnostic competence can be developed through structured professional learning activities that require teachers to examine student responses, compare alternative interpretations, justify their judgments with evidence, and consider possible instructional responses (Prediger & Zindel, 2017). Such activities may help pre-service teachers move beyond evaluating whether a solution is correct and toward analyzing the mathematical reasoning that underlies students’ responses.

Research suggests that the development of diagnostic competence depends not only on teachers’ professional knowledge but also on the learning opportunities and support available to them. In particular, learners with different levels of prior knowledge may benefit differently from instructional scaffolding (Chernikova et al., 2020). This suggests that individual differences in pre-service teachers’ initial diagnostic competence may be relevant to understanding how they engage with and benefit from professional learning activities.

Although previous research has examined teachers’ interpretations of observable student thinking, less is known about how pre-service mathematics teachers anticipate possible student strategies, errors, and mathematical understandings before actual student responses are available. The present study addresses this issue by examining AI-supported mathematical task design as a context for developing pre-service teachers’ understanding of student mathematical thinking. The theoretical perspectives on mathematical task design and AI-supported learning are discussed in the following sections.

### 2.2. Mathematical Task Design

Mathematical tasks are central to mathematics teaching and learning because they provide opportunities for students to engage with mathematical ideas and shape the nature of their mathematical activity (Doyle & Carter, 1984; Smith & Stein, 1998). Task design therefore involves more than selecting mathematical content or producing a problem statement. It requires teachers to coordinate mathematical goals, task characteristics, and anticipated student activity. Research on teachers’ professional knowledge suggests that designing mathematical tasks draws on both mathematical and pedagogical knowledge, including knowledge of mathematical concepts, students’ prior knowledge, possible solution strategies, and potential difficulties (Loewenberg Ball et al., 2008; Baumert et al., 2010; Alsina et al., 2024; Kara & Corum, 2022).

Importantly, task design requires teachers to reason about how students may engage with the mathematics embedded in a task. Teachers may need to anticipate possible solution strategies, errors, misconceptions, and differences in students’ mathematical understanding. In this sense, task design can create opportunities for teachers to make prospective judgments about student mathematical thinking before actual student responses are available. Such prospective reasoning is closely related to the diagnostic thinking described in Section 2.1, particularly the processes of attending to relevant features, interpreting possible student thinking, and forming judgments about students’ mathematical understanding. Research on task design and teacher learning further suggests that engaging teachers in the design, analysis, and revision of mathematical tasks can provide opportunities to develop and mobilize professional knowledge (Xu & Brown, 2016; Dempsey & O’Shea, 2020).

Nevertheless, existing research has more often examined the characteristics and quality of designed tasks or the development of teachers’ general professional knowledge than the extent to which task design specifically develops teachers’ ability to understand students’ mathematical thinking. Moreover, little is known about how prospective mathematics teachers engage in such reasoning when supported by emerging technologies. These limitations motivate the present study, which examines AI-supported mathematical task design as a context for developing pre-service mathematics teachers’ ability to understand student mathematical thinking. The role of artificial intelligence as a learning tool is discussed in the following section.

### 2.3. Artificial Intelligence as a Learning Tool

Recent advances in generative artificial intelligence have expanded the role of AI in education from technologies that deliver predefined content to interactive tools that can generate responses, provide feedback, and support learners’ iterative engagement with knowledge (Lameras & Arnab, 2021; Tan et al., 2025). In teacher education, AI has increasingly been explored as a tool for supporting professional learning and teaching-related activities, including the development of instructional materials, lesson planning, and pedagogical decision-making (Besimi et al., 2022; Lo, 2023; Cai et al., 2025; Uygun et al., 2025). From a learning perspective, the potential value of AI lies not only in the information it provides but also in the opportunities it creates for learners to explore alternatives, obtain feedback, and revise their thinking through iterative interaction. Such affordances may be particularly relevant to professional learning activities that require teachers to consider multiple possible interpretations of mathematical content and student thinking.

Human–AI interaction is shaped substantially by how learners formulate and refine their prompts. The content of prompts influences the information and responses generated by AI and may therefore shape the subsequent learning interaction (M. Liu et al., 2024; H. Nguyen & Nguyen, 2025; Kim, 2026). At the same time, prompts can provide observable traces of learners’ intentions, questions, information-seeking strategies, and engagement with professional knowledge during interaction with AI (A. Nguyen et al., 2024; Guo et al., 2025). From this perspective, prompt data may provide a useful source of evidence for examining how pre-service teachers engage with AI and mobilize professional knowledge during learning activities. In the present study, prompts are therefore analyzed as observable indicators of participants’ interaction with AI rather than as direct measures of cognition.

Learners’ perceptions of AI may also influence their engagement with AI-supported learning. Research based on technology acceptance models suggests that perceived usefulness and related acceptance beliefs are associated with users’ intentions to adopt and use educational technologies (Nikolopoulou et al., 2020). Recent research on AI in education has similarly highlighted the importance of users’ attitudes and acceptance in shaping AI adoption and use (Cai et al., 2025; Wang et al., 2025). In the present study, AI acceptance is therefore considered as an individual-level condition that may be relevant to how pre-service teachers engage with AI-supported learning activities. Together with participants’ prompting behavior and initial diagnostic competence, it is examined as part of a configurational analysis of factors associated with learning outcomes.

## 3. Methodology

### 3.1. Participants

All participants had completed their secondary and undergraduate education within the Chinese educational system and were enrolled in postgraduate programs at the time of the study. They had received prior training in mathematics and mathematics education, including coursework in areas such as mathematics curriculum and instruction, and were preparing for careers as mathematics teachers. Participants also had varying levels of practical experience in school settings: 16 had completed at least one educational internship, and 5 had prior formal teaching experience. A preliminary survey indicated that all participants had prior experience using AI tools and were familiar with basic AI platform operations, although their frequency of AI use varied. Participants were recruited from a mathematics teacher education program, and participation was voluntary. Before data collection, all participants provided informed consent for participation and the use of their data for research purposes. Although the intervention was integrated into the teacher education course, participation in the research component did not influence course grading. The intervention consisted of nine sessions implemented over six weeks within a ten-week teacher education course. During the intervention, participants engaged in an iterative cycle of mathematical task design, AI prompt formulation, interaction with the DeepSeek AI, task revision, and reflection.

### 3.2. Research Design

**Figure 1:**
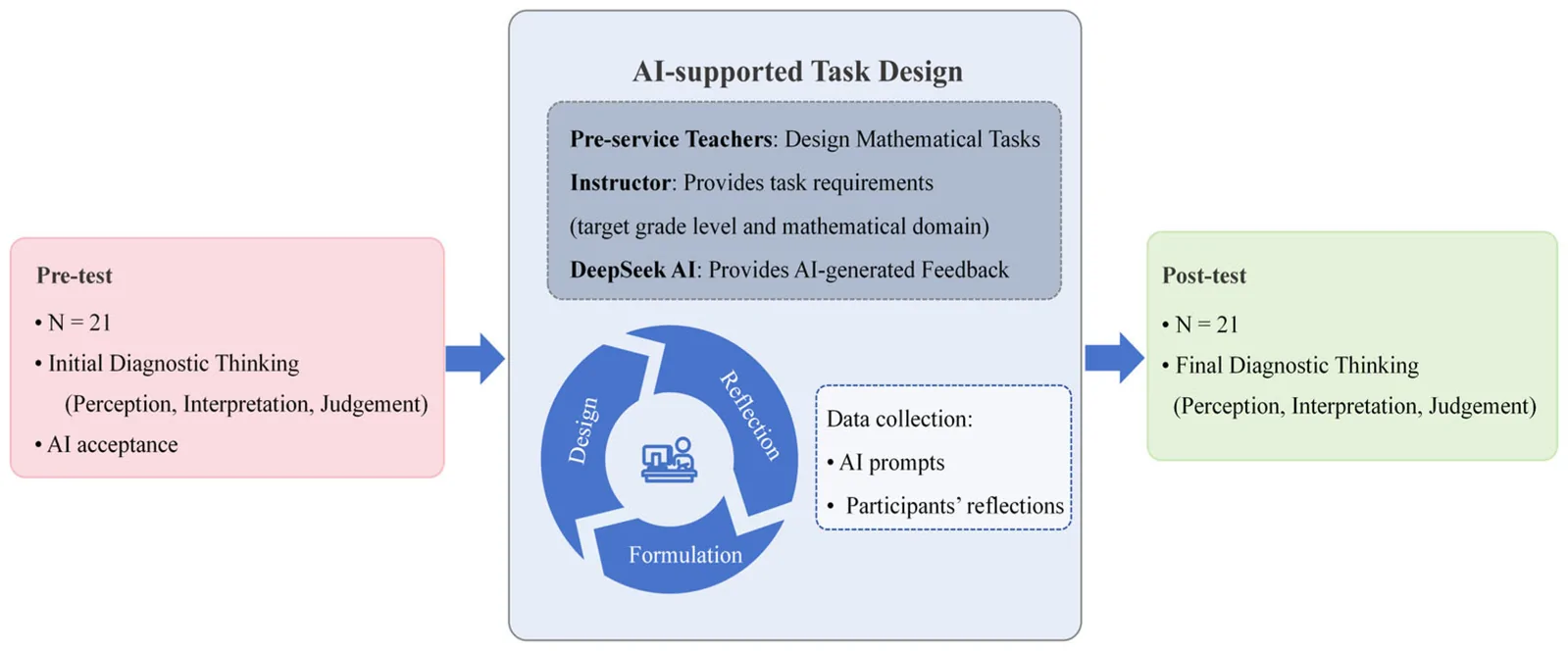
Research procedure and data collection.

**Figure 2:**
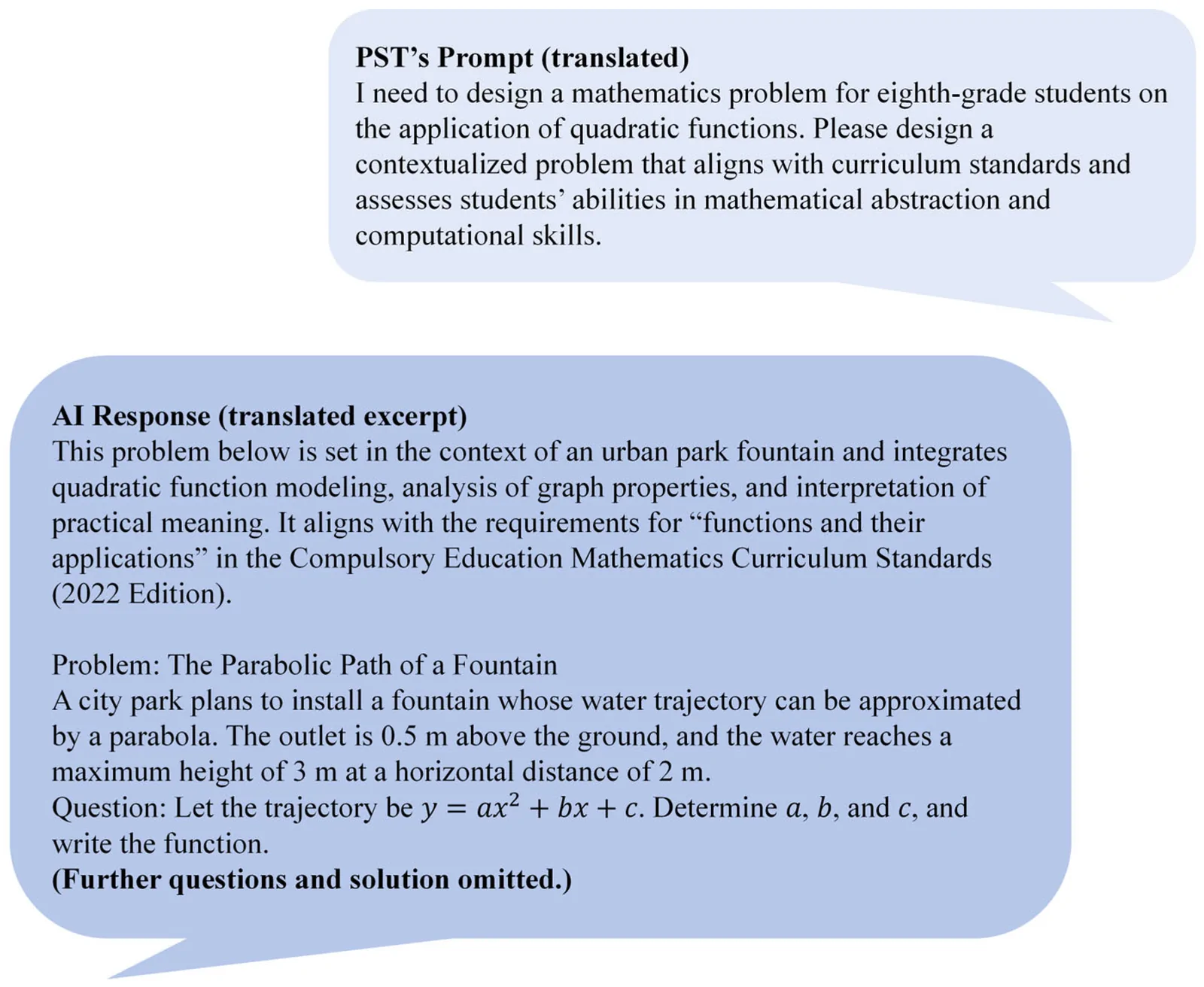
The interface of the DeepSeek platform.

The intervention consisted of a six-week module comprising nine instructional sessions. The course process was conducted by the researcher. Participants engaged in three iterative task design cycles, with each cycle focusing on a different mathematical content domain: geometry, algebra, and statistics. Each cycle consisted of three phases—design, reflection, and formulation (Figure 1). All participants used the same version of the DeepSeek AI platform (Version 3), with a standardized interface and default settings to minimize variability attributable to the AI tool. The platform enabled natural-language interaction and generated real-time responses, allowing participants to obtain information, alternative solutions, explanations, and feedback relevant to mathematical content and pedagogical considerations (Figure 2).

During the design phase, participants collaborated with AI to develop a mathematical task for the designated content domain. In this study, task design refers to the process of creating mathematical problems for students to solve. All tasks were required to be situated in authentic or realistic contexts and aligned with the cognitive demands appropriate for the target grade level. Participants were also asked to consider relevant mathematical concepts, possible student strategies and misconceptions, and the anticipated difficulty of the task in relation to students’ prior knowledge. Participants interacted iteratively with AI to generate, examine, and refine their tasks. All prompts and AI-generated responses were automatically recorded for subsequent analysis.

During the reflection phase, participants documented their immediate reflections on the task design process and their interactions with AI. They were asked to consider how AI-generated responses informed their design decisions, where AI-generated suggestions required modification or correction, and how the task could be revised to better support the intended cognitive demands and anticipated student thinking. During the formulation phase, participants consolidated their reflections and produced a final version of the task. The same three-phase process was repeated across the three content domains, enabling participants to engage in iterative cycles of AI-supported task design, reflection, and task formulation.

### 3.3. Data Collection and Measurement

As shown in Figure 1, the study examined three configurational conditions representing psychological, competence-related, and behavioral aspects of participants’ engagement with the AI-supported learning process: (1) AI acceptance, reflecting participants’ perceptions and attitudes toward the use of AI; (2) initial diagnostic thinking, measured through the pre-intervention assessment and representing participants’ baseline competence; and (3) AI prompt content, coded as an observable indicator of participants’ cognitive engagement and activation of relevant professional knowledge during human–AI collaboration. The post-intervention assessment of diagnostic thinking served as the outcome variable.

The study used these conditions to examine how different combinations of participants’ initial competence, AI acceptance, and interactional behavior were associated with high levels of post-intervention diagnostic thinking. This configurational perspective allowed the study to identify multiple configurations linked to higher levels of diagnostic thinking to understand students’ mathematical thinking during AI-supported task design.

#### 3.3.1. Diagnostic Thinking

Diagnostic thinking was assessed across three dimensions: (1) Perception, (2) Interpretation, and (3) Judgement, following the cognitive process described in prior research (Brunner et al., 2024; Leuders et al., 2018, pp. 125–126). Two alternate paper-and-pencil assessment forms, Form A and Form B, were developed using mathematical problems drawn from China’s National College Entrance Examination test papers (Beijing Gaokao test papers and Gaokao Mock test paper; see the test forms in Appendix A and Appendix B). Each form included one solid geometry problem and one trigonometry problem and followed a comparable open-ended task structure, with the same assessment structure and scoring framework. The problems were selected based on their alignment with the assessment framework and their potential to elicit PSTs’ diagnostic reasoning. Available student-performance data from the original examination contexts showed indices ranging from 0.80–0.89. Forms A and B were designed to be similar in mathematical content domains, task structure, and diagnostic prompts, with both requiring PSTs to analyze mathematical conditions, anticipate student difficulties, and provide diagnostic judgments. Importantly, the problems served as diagnostic stimuli rather than measures of participants’ own mathematical achievement. The diagnostic prompts following the mathematical problems were identical across the two forms, ensuring that PSTs engaged in the same diagnostic processes across measurement occasions.

To further reduce potential effects associated with test form differences and administration order, the order of administration was counterbalanced across participants. Specifically, 10 participants completed Form A at pre-test and Form B at post-test (AB sequence), whereas 11 participants completed Form B at pre-test and Form A at post-test (BA sequence). Participants were randomly assigned to the two sequences. Thus, every participant completed both forms, but the association between test form and measurement occasion was reversed across the two groups. To examine whether the observed pre–post changes were associated with administration sequence, change scores were compared between the AB and BA groups using an independent-samples *t*-test. The mean change score was 4.40 (SD = 3.44) for the AB group and 5.00 (SD = 3.22) for the BA group. The difference in change scores between the two groups was not statistically significant, t(19) = −0.41, *p* = .684, mean difference = −0.60, 95% CI [−3.64, 2.44]. Given the small group sizes, however, this result should be interpreted cautiously and should not be taken as evidence that Forms A and B were equivalent.

To operationalize the three dimensions, participants were asked to complete three types of diagnostic tasks. Following each mathematical problem, three diagnostic prompts were provided to explicitly elicit the three dimensions of diagnostic thinking. First, they identified the prerequisite mathematical knowledge and potential sources of difficulty associated with each problem, providing evidence of Perception. Second, they identified potential student difficulties and explained the mathematical reasons underlying these difficulties, providing evidence of Interpretation. Third, they estimated the expected success rate of the target student group and explained the basis for their estimates, providing evidence of Judgement. The target students were defined as Grade 12 students. For the Judgement dimension, reference success rates were established by the first author, who has extensive experience in high-stakes mathematics assessment development, using available student-performance data from the original examination contexts as empirical benchmarks. Across the selected problems, the reference success rates ranged from 0.80 to 0.89. Item-level reference rates are not reported separately because item-level performance statistics from these high-stakes assessment materials are not publicly reportable. PSTs’ estimates were evaluated according to their absolute difference from these reference rates. Scores were summed across the two mathematical problems, yielding possible score ranges of 0–6 for Perception, 0–6 for Interpretation, and 0–4 for Judgement, and an overall diagnostic thinking score ranging from 0 to 16.

Participants’ written responses were coded using a qualitative content analysis framework informed by Brunner et al. (2021) and Loibl et al. (2020). Detailed scoring criteria and illustrative examples for each dimension are provided in Appendix C. Two independent raters coded all responses, and inter-rater reliability was assessed using a two-way random-effects intraclass correlation coefficient with absolute agreement for average measures (ICC (2,k) = 0.95). Any discrepancies were resolved through discussion until consensus was reached.

#### 3.3.2. Acceptance of AI

Before the intervention, all 21 participants completed a survey assessing their acceptance of AI. The instrument was developed based on the Unified Theory of Acceptance and Use of Technology (UTAUT) framework (Nikolopoulou et al., 2020; Williams et al., 2015). It consists of 25 items (see part of the survey in Appendix D), all rated on a five-point Likert scale ranging from 1 (strongly disagree) to 5 (strongly agree). The scale demonstrated high internal consistency (Cronbach’s α = 0.916).

#### 3.3.3. Knowledge Density in AI Prompts

This study focused on how pre-service teachers activated their Mathematical Knowledge for Teaching (MKT) during human–AI collaboration rather than on the technical quality of their prompting strategies. Accordingly, the content of participants’ prompts was analyzed to characterize the extent and nature of professional knowledge activated during AI-supported task design. All chatlogs generated during the intervention were collected, yielding a total of 438 prompts.

To examine how participants activated professional knowledge in AI interactions, we conducted a theory-informed thematic analysis (Braun & Clarke, 2006). Content Knowledge (CK) and Pedagogical Content Knowledge (PCK), based on the Mathematical Knowledge for Teaching framework (Loewenberg Ball et al., 2008), were used as theoretical lenses for developing the coding framework. We developed a deductive coding scheme to distinguish prompts by their incorporation of mathematical and pedagogical knowledge. During the iterative coding, an additional data-driven category (d) emerged from the prompt data. The final scheme comprised four ordinal prompt types representing increasing levels of professional knowledge density (Table 1). Two independent raters coded all prompts. Inter-rater reliability was assessed using a two-way random-effects intraclass correlation coefficient with absolute agreement for average measures (ICC (2,k) = 0.92), and discrepancies were resolved through discussion and consensus.

Knowledge Density in AI Prompts (PKD) was calculated as the average score of all prompts produced by each participant during the intervention. Specifically, PKD was calculated using the following formula:

(1) $${PKD}_{i}=\frac{\sum_{j=1}^{n_{i}}S_{ij}}{4n_{i}},$$

where *PKD_i_* represents the knowledge density score of participant *i*, *S_ij_* represents the coding score (1–4) assigned to the *j*-th prompt generated by participant *i*, and *n_i_* represents the total number of prompts generated by participant *i*. Dividing by the maximum possible score (4) transformed the indicator into a standardized value ranging from 0.25 to 1. Higher PKD values indicate that a greater proportion of participants’ AI interactions involved prompts incorporating deeper mathematical and pedagogical knowledge considerations.

## 4. Data Analysis

### 4.1. Paired-Samples t-Test

Quantitative analyses were conducted to address RQ1. Paired-samples *t*-tests were used to compare PSTs’ diagnostic thinking scores before and after the six-week intervention. Specifically, we examined pre–post differences in the overall diagnostic thinking score and in each of its three dimensions: Perception, Interpretation, and Judgement.

### 4.2. Fuzzy-Set Qualitative Comparative Analysis

To address RQ2, fuzzy-set Qualitative Comparative Analysis (fsQCA) was used to examine how different combinations of configurational conditions were associated with high levels of post-intervention diagnostic thinking. The analysis was conducted using fsQCA 4.1 software. The analysis involved data calibration, truth table construction, configurational analysis, and subsequent robustness checks.

First, the measured variables were calibrated and transformed into fuzzy-set membership scores ranging from 0 to 1 (Schneider & Wagemann, 2012, pp. 52–53). Calibration anchors can be determined based on theoretically or empirically informed criteria (Ragin, 2008; Schneider & Wagemann, 2012). Given the exploratory nature of this study, an empirical distribution-based calibration approach was adopted to identify configurational patterns within the observed sample. Because pre-test and post-test diagnostic thinking were treated as occasion-specific fuzzy sets (i.e., IDT and FDT), calibration thresholds were determined separately for each measurement occasion based on the empirical distribution of the corresponding scores. Following established fsQCA procedures (Rihoux & Ragin, 2009), three calibration anchors were specified for each fuzzy set: full membership, the crossover point, and full non-membership. These corresponded to membership scores of 0.95, 0.50, and 0.05, respectively. The corresponding raw-score anchors should be interpreted as sample-informed reference points rather than externally established cutoffs. Substantively, higher membership in the IDT and FDT sets indicates relatively stronger diagnostic thinking performance, higher membership in the ACC set indicates greater acceptance of AI, and higher membership in the PKD set indicates a greater predominance of prompts incorporating mathematical and pedagogical knowledge. Inspection of the raw distributions showed that the numbers of cases at or beyond the lower and upper outer anchors were 2 and 5 for IDT, 3 and 3 for ACC, 2 and 2 for PKD, and 2 and 4 for FDT, respectively. Thus, each outer calibration anchor was supported by at least two cases rather than by a single observation. For example, in the pre-test, participants scoring 12 or above on the diagnostic thinking assessment were calibrated as having a high degree of membership in the set of participants with high initial diagnostic thinking, whereas those scoring 2 or below were calibrated as having a high degree of non-membership in this set. The raw-score thresholds used for calibration are reported in Table 2.

The data were calibrated using the calibrate (x, n1, n2, n3) function in fsQCA 4.1. Table 2 presents the calibration anchors and their substantive interpretations for the three configurational conditions and the outcome set examined in this study. After calibration, cases with membership scores exactly equal to 0.50 were adjusted to 0.501 by adding a constant of 0.001 before truth table construction to avoid ambiguity at the crossover point (Fiss, 2011; Cangialosi, 2023).

Next, necessity analysis was conducted to examine whether any individual configurational condition was necessary for the outcome (Ragin, 2008). A condition was considered necessary when its consistency exceeded 0.90. We then constructed a truth table using a consistency threshold of 0.80 and a proportional reduction in inconsistency (PRI) threshold of 0.70 (J. Liu et al., 2024). The analysis generated three types of solutions: parsimonious, intermediate, and complex. Following established fsQCA practice, the intermediate solution was used as the primary basis for interpreting the configurations, while the parsimonious solution was used to identify core conditions (Ragin, 2008). Finally, robustness checks were conducted by varying key analytical parameters. The resulting configurations were compared with the main analysis to assess the stability of the identified configurational patterns.

### 4.3. Qualitative Analysis

To address RQ3, we conducted a thematic analysis of participants’ reflections and AI interaction records, including their prompts and the corresponding AI responses. All participants’ prompts were included in the thematic analysis. Because PKD was already included as an fsQCA condition, the qualitative analysis focused on interactional processes beyond prompt density, particularly how PSTs engaged with, evaluated, and revised AI-generated suggestions. Following Braun and Clarke’s (2006) approach, the data were coded to identify recurring interactional processes. For each high-FDT configuration, we identified all cases with membership above 0.50 in every element of the configuration and in the corresponding outcome. This procedure identified two cases for H1 and three cases for H2, contributing 43 and 63 prompts, respectively. For these five cases, participants’ reflections and AI interaction records were examined alongside the coded prompts to identify recurring sequences of AI response, participant evaluation, and subsequent revision. Illustrative excerpts were then selected from the five cases to represent these recurring interactional processes, particularly sequences that clearly showed how participants evaluated and acted on AI-generated suggestions. Accordingly, the detailed process analysis used to address RQ3 focused on these five configuration-associated cases rather than on the full sample of 21 participants. The resulting patterns are therefore interpreted as illustrative case-based observations rather than as broader differences between H1 and H2.

## 5. Results

### 5.1. Changes in PSTs’ Diagnostic Thinking During the Module

To address RQ1, paired-samples *t*-tests were conducted to examine changes in PSTs’ diagnostic thinking following the six-week module. Prior to the analyses, the normality assumption of the difference scores was assessed using the Shapiro–Wilk test. The results indicated that none of the difference scores significantly deviated from normality (all *p*s > .05). Table 3 presents the results of the paired-samples *t*-tests for the overall diagnostic thinking score and its three dimensions: Perception, Interpretation, and Judgement.

**Figure 3:**
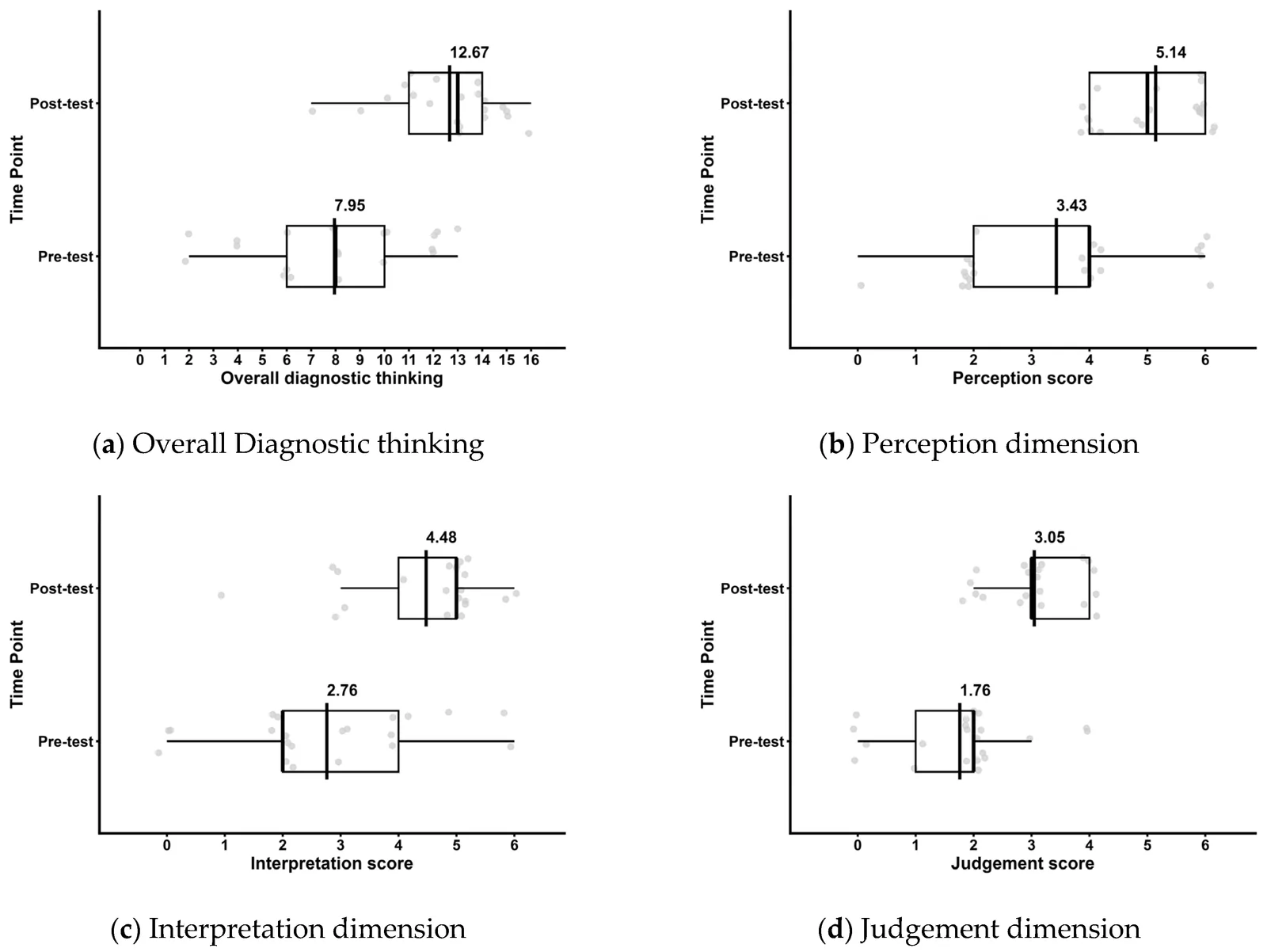
Distribution of scores across the three dimensions and overall diagnostic thinking. Individual student scores are represented by jittered scatter points to ensure clarity. The boxplots illustrate the interquartile range, while the bold vertical lines and accompanying numerical values denote the mean and median scores for the pre-test and post-test, respectively.

Overall diagnostic thinking (DT) scores increased from the pre-test (M = 7.952) to the post-test (M = 12.667), representing a mean increase of 4.714 points. This difference was statistically significant, t(20) = 6.631, *p* < .001. The result indicates scores of participants’ diagnostic thinking were significantly higher after the six-week task design intervention (see Figure 3a).

Improvements were also observed across all three dimensions of diagnostic thinking. Scores on Perception increased from a pre-test mean of 3.429 to a post-test mean of 5.143, t(20) = 4.315, *p* < .001 (see Figure 3b). This suggests that participants demonstrated stronger performance in attending to and identifying relevant features of mathematical tasks. Scores on Interpretation increased significantly from 2.762 to 4.476, t(20) = 4.954, *p* < .001 (see Figure 3c), indicating improved performance in interpreting potential student difficulties and the reasoning underlying students’ possible mathematical responses. Scores on Judgement increased from 1.762 to 3.048, t(20) = 4.258, *p* < .001 (see Figure 3d), suggesting improvement in participants’ ability to evaluate the likely difficulty of mathematical tasks for students.

As shown in the boxplots (Figure 3), post-test scores were generally higher than pre-test scores in terms of both means and medians. The distributions of individual scores also shifted upward overall, although the extent of improvement varied across participants. These patterns are consistent with the significant pre–post differences reported above. Because initial diagnostic thinking was subsequently included as a configurational condition, we also examined the association between the pre- and post-test overall diagnostic thinking scores. The association was modest and positive, *r*(19) = 0.36, *p* = 0.105. To further examine this heterogeneity, we next examined whether different combinations of conditions were associated with high post-intervention diagnostic thinking.

### 5.2. Configurational Pathways to High Post-Intervention Diagnostic Thinking

Before identifying sufficient configurations, we first examined whether any single condition was necessary for high post-intervention diagnostic thinking. In fsQCA, a condition is generally considered necessary when its consistency exceeds 0.90. The results of the necessity analysis are presented in Table 4. The consistency values for all individual conditions were below 0.75, indicating that no single condition was necessary for the outcome. This finding suggests that high levels of post-intervention diagnostic thinking were not attributable to any single condition alone but may instead be associated with different combinations of conditions.

Given that the analysis included three configurational conditions, the truth table contained eight (2^3^ = 8) possible logical configurations. Following the specified frequency cutoff of 1 and PRI consistency threshold of 0.70 (Patala et al., 2021; Ragin, 2008), the complete truth table was evaluated to identify configurations associated with high diagnostic thinking. The complete truth table, including configuration rows, case frequencies, raw consistency, PRI consistency, and outcome assignment, is reported in Supplementary Tables S1 and S2.

The fsQCA generated complex solutions, parsimonious solutions, and intermediate solutions. For the intermediate solution, directional expectations were specified to guide the identification of theoretically plausible configurations. Specifically, the presence of IDT, ACC, and PKD was expected to contribute positively to high FDT, whereas their absence was considered relevant for identifying configurations associated with low FDT. The intermediate and parsimonious solutions yielded identical configurations; therefore, all conditions reported in Table 5 were identified as core conditions, with no peripheral conditions distinguished. As shown in Table 5, two sufficient configurations (H1 and H2) were associated with high post-intervention diagnostic thinking. Their overall solution consistency was 0.853, and the overall solution coverage was 0.468, indicating that the two configurations together covered 46.8% of the empirical membership in the outcome set. This relatively limited coverage suggests that other pathways to high post-intervention diagnostic thinking may remain unexplained by the conditions examined in this study. One sufficient configuration (L1) was identified for low post-intervention diagnostic thinking, with a consistency of 0.931 and a coverage of 0.400. Thus, L1 showed a high degree of consistency with the outcome and covered 40.0% of the empirical membership in the low post-intervention diagnostic thinking set. In fsQCA, consistency reflects the extent to which a configuration is reliably associated with an outcome, whereas coverage indicates the empirical relevance of the configuration in accounting for that outcome (Pappas & Woodside, 2021).

**Figure 4:**
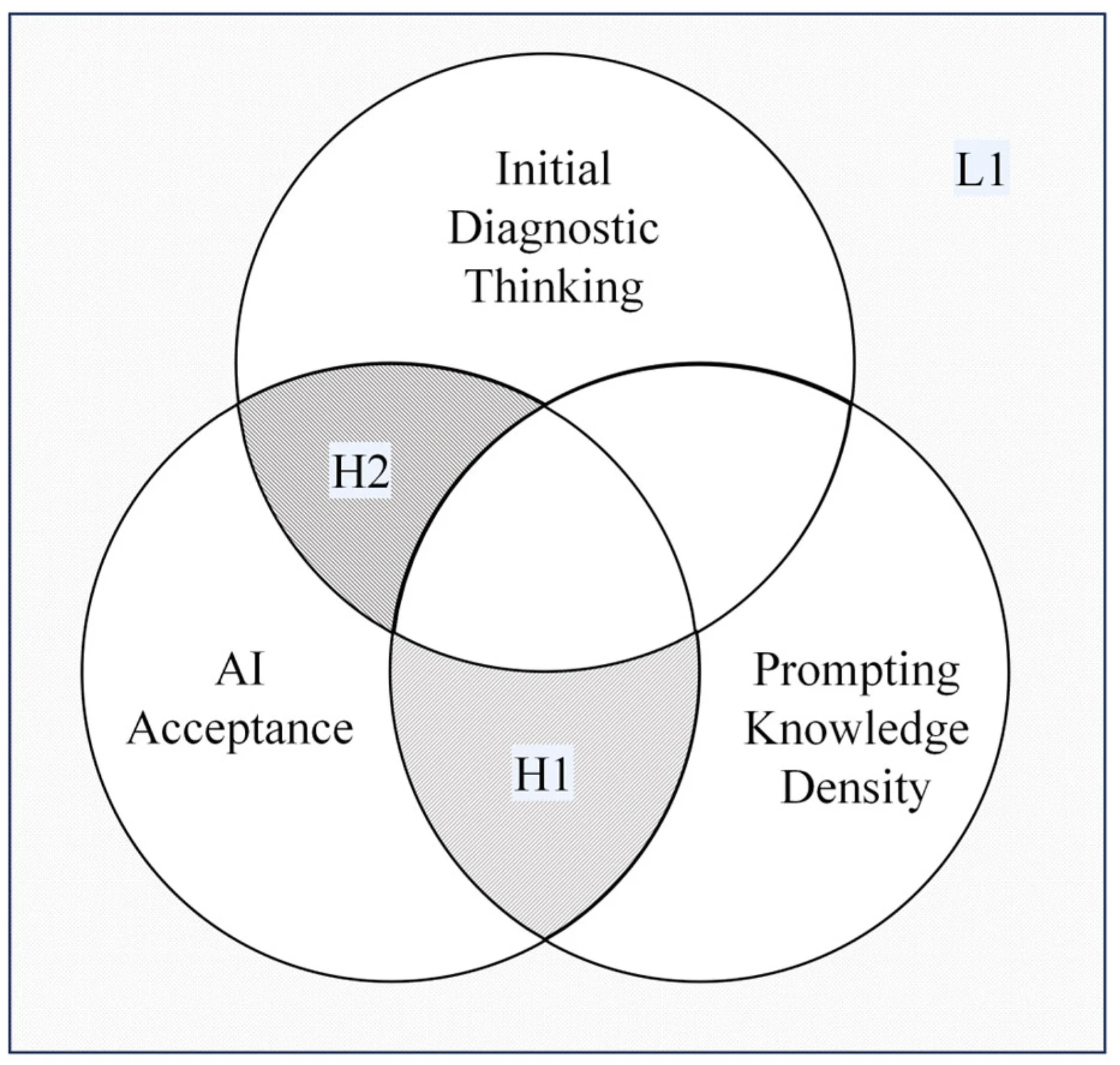
Three configurations identified in the fsQCA.

Figure 4 illustrates the three configurations identified in the fsQCA. High post-intervention diagnostic thinking was associated with either high AI acceptance combined with high prompting knowledge density despite low initial diagnostic thinking (H1: ~IDT*ACC*PKD) or high initial diagnostic thinking and AI acceptance despite low prompting knowledge density (H2: IDT*ACC*~PKD). Low post-intervention diagnostic thinking was associated with low levels of all three conditions (L1: ~IDT~ACC~PKD).

To examine the robustness of the configurational analysis results, sensitivity analyses were conducted by modifying key parameters. The detailed results of these robustness checks are reported in Supplementary Table S3. First, the calibration thresholds were adjusted from 95%, 50%, and 5% to 90%, 50%, and 10%, respectively. Under this alternative specification, H1 and L1 remained unchanged, whereas H2 was not identified. Second, increasing the frequency cutoff from 1 to 2 did not alter the identified configurations, with H1, H2, and L1 all retained. Third, adjusting the raw consistency threshold to 0.82 yielded the same three configurations, indicating that the results were not sensitive to minor changes in the consistency criterion. Finally, increasing the PRI consistency threshold from 0.70 to 0.75 retained H1 and L1, whereas H2 disappeared. Overall, the robustness analysis indicates that the configurations associated with high and low final diagnostic thinking were generally stable across alternative specifications. However, the sensitivity of H2 to alternative calibration thresholds and PRI criteria suggests that this configuration should be interpreted with caution and treated as exploratory, particularly given the pilot nature and small sample size of this study.

### 5.3. Interaction Patterns Across Configurational Pathways

To address RQ3, we first examined the thematic patterns in participants’ AI prompts using the established prompt-coding framework (Types A–D). We then related these prompt themes to participants’ reflections and interaction records to examine how they evaluated AI-generated suggestions and how those evaluations informed subsequent revisions. The following process-oriented analysis is based on the five configuration-associated cases (two H1 and three H2 cases) and aims to illustrate how these PSTs engaged with, evaluated, and revised AI-generated suggestions, rather than to establish broader differences between H1 and H2.

**Figure 5:**
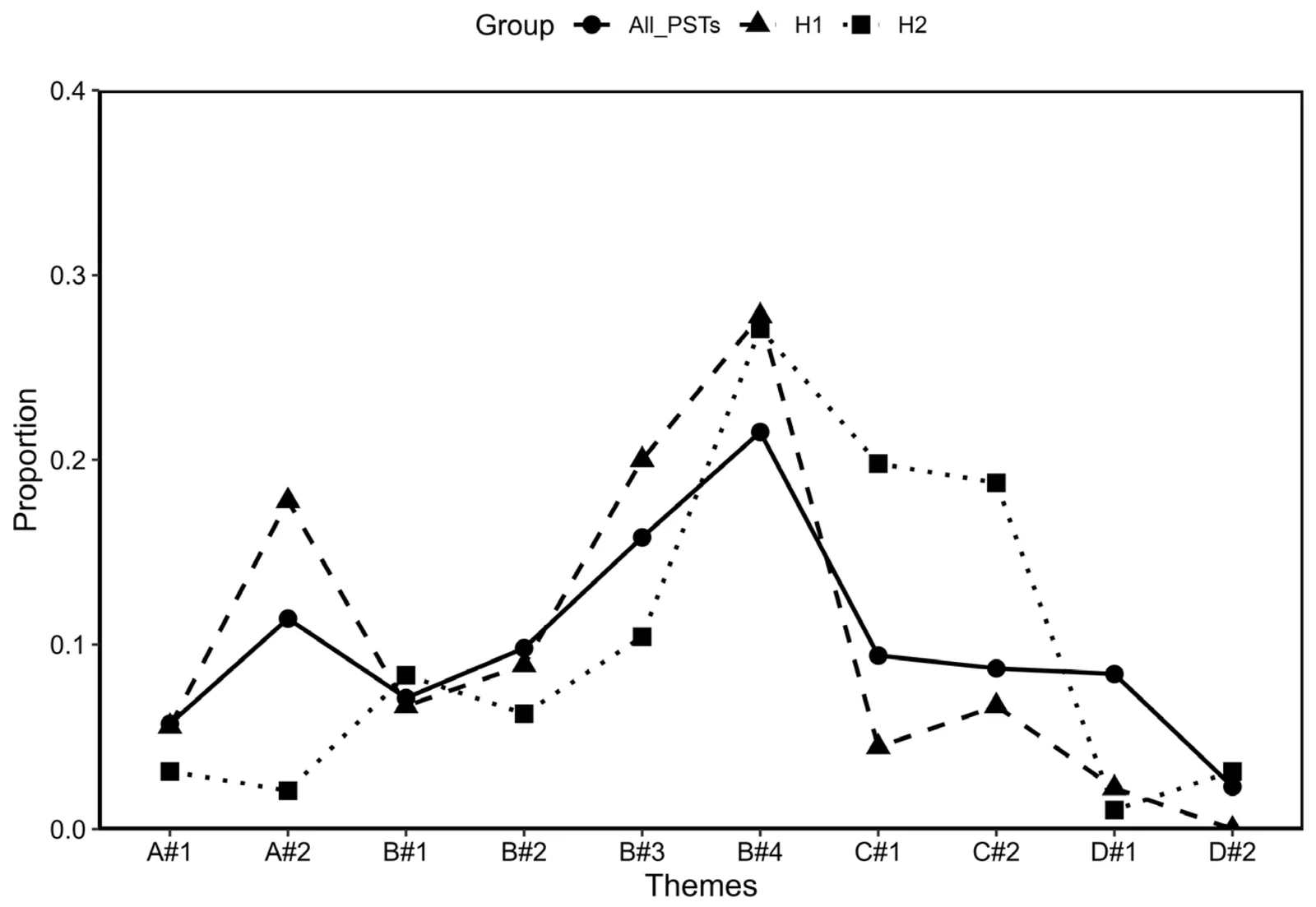
PSTs’ AI Prompts Themes and Proportion.

Ten sub-dimensions were identified across the four types, and their frequencies and percentages are presented in Table 6 and Figure 5. Across all participants, prompts most frequently addressed task difficulty adjustment (B#3), curriculum alignment (B#4), and task-context validity (A#2). By contrast, prompts involving AI-assisted self-checking and general task revision (D#2 and D#1) were less frequent. The pattern indicates that prompts related to cognitive demand and content alignment were more common than those focused on general task revision.

**Figure 6:**
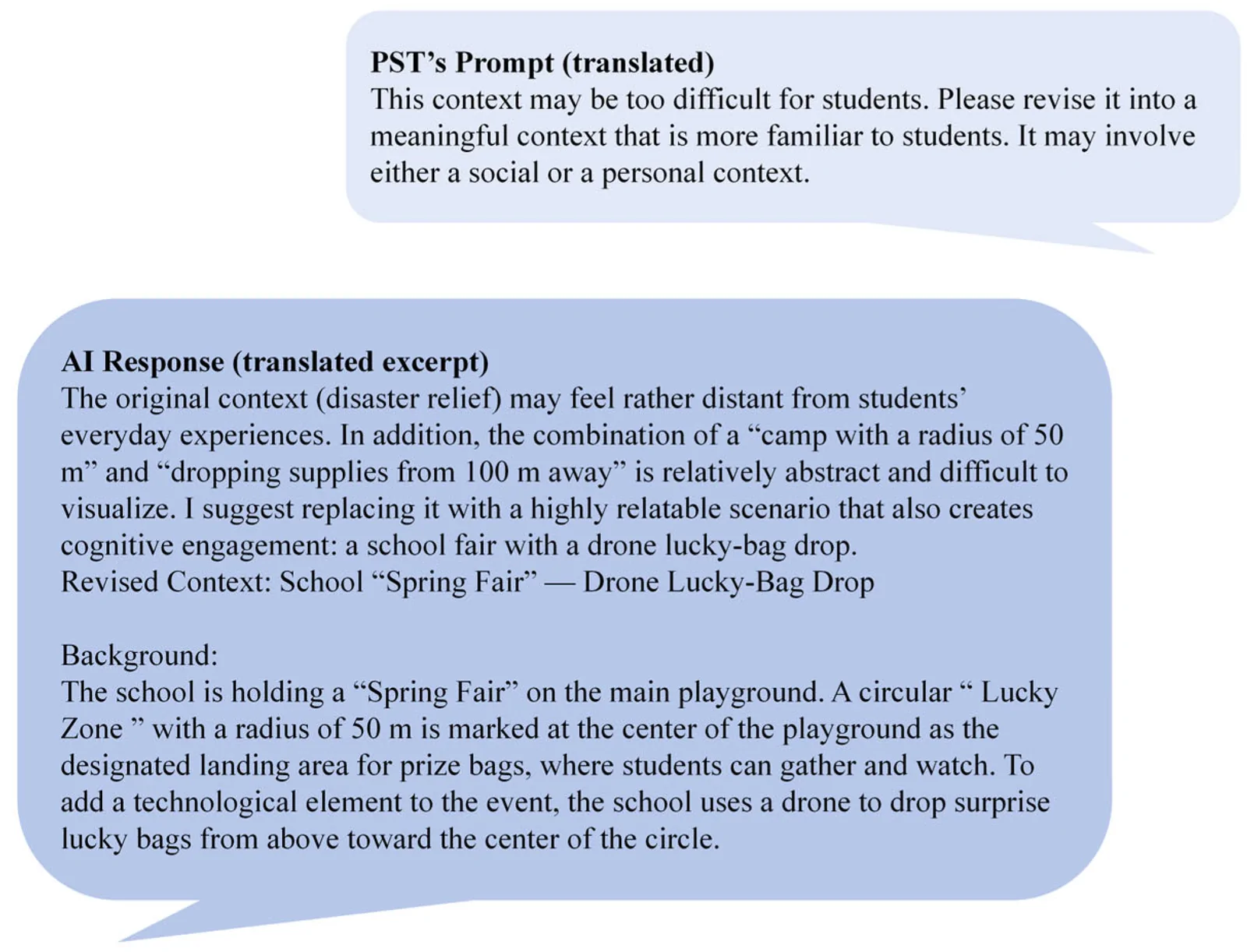
An example of A#2.

The H1 cases (~IDT*ACC*PKD) more often used prompts reflecting Integrated PCK–CK Focus, particularly attention to task context (A#2), and Isolated Knowledge Focus, particularly adjustment of task difficulty to grade level (B#3), than the overall sample. One participant, for example, expressed a tentative concern about the suitability of the task context, prompting the AI: “This context may be too difficult for students. Please revise it into a meaningful context that is more familiar to students.” (A#2; see Figure 6). In the subsequent reflection, the participant explained: “The original scenario was confusing for students, so I revised it to something familiar and meaningful, ensuring they could interpret the problem logically.” Taken together, the prompt and reflection show that the participant first articulated a tentative concern about possible student difficulty and then used this concern to guide revision of the task context. H1 prompts from another participant also included requests for the AI to assess whether tasks were appropriate for the intended grade level, such as: “Does your question meet the ability requirements for eighth-grade students? Please make the necessary modifications” (B#3). In these examples, concerns about student understanding or task difficulty were sometimes expressed tentatively or as questions to the AI rather than as fully specified revision instructions. Given the relatively lower initial diagnostic thinking levels represented in H1, this question-oriented interaction suggests that AI was used partly as a resource for checking emerging judgements about students and task appropriateness before participants decided how to revise the task.

The H2 cases (IDT*ACC*~PKD), in contrast, tended to use more selective and targeted prompts focused on particular aspects of task design and presentation. Compared with the overall sample, these cases showed relatively higher engagement with C#1 and C#2, concerning task readability, accessibility, and task format or illustration, but lower engagement with A#2, concerning task context. For example, one participant repeatedly asked the AI to generate a more precise diagram with point M on line l (C#2), explaining in the reflection: “I requested the AI three consecutive times to generate a more precise diagram because the most time-consuming part of task design is creating figures.” In this exchange, AI served a task-specific supportive role by assisting with figure generation, which the participant regarded as the most time-consuming part of task design. Another participant noted that some AI-generated problems were “sometimes too difficult or not sufficiently rigorous.” She subsequently provided more specific constraints to the AI, including: “The curriculum does not require knowledge of derivatives; please remove this content” (B#4). Here, the participant evaluated the AI-generated task against curriculum requirements and translated that evaluation into a specific revision instruction. The interaction therefore involved a clearer response–evaluation–revision sequence, in which a problem in the AI-generated task was identified, evaluated against a professional criterion, and addressed through a more specific subsequent instruction.

Across these cases, the qualitative evidence extends beyond differences in prompt composition by showing how participants engaged with and acted on AI-generated responses. In the H1 cases, interactions were sometimes framed as questions or tentative requests for evaluation, suggesting that AI was used partly to check emerging judgements about student understanding and task appropriateness. In the H2 cases, interactions more often involved identifying a specific problem and issuing a targeted revision instruction, with AI also serving task-specific functions such as figure generation. These observations suggest that the two configurations differed not only in the knowledge reflected in participants’ prompts but also in how AI was used during task evaluation and revision.

## 6. Discussion

### 6.1. Changes in Diagnostic Thinking During the AI-Supported Task Design Module

The significantly higher post-test scores in overall diagnostic thinking and its three dimensions indicate that participants’ performance changed over the six-week module. Given the single-group pre–post design, these changes cannot be attributed specifically to AI-supported task design or to any individual component of the module. Rather, the module provided repeated opportunities for PSTs to engage in diagnostic reasoning during mathematical task design. In particular, PSTs examined the mathematical structure of tasks, anticipated possible student difficulties, and judged task appropriateness for particular learners and curricular expectations, thereby engaging in processes corresponding to perception, interpretation, and judgement before actual student responses were available.

One possible explanation for the observed pattern is that mathematical task design itself has a diagnostic character. Designing a task requires teachers to identify the mathematical knowledge involved, anticipate how students might approach the task, consider possible misconceptions, and evaluate the level of cognitive demand and difficulty. In this sense, task design may provide opportunities for teachers to reason about students’ possible mathematical thinking before actual student responses are available. This extends previous research that has primarily examined teachers’ understanding of student thinking through existing student work, classroom interactions, or other observable evidence (Loibl et al., 2020; Rieu et al., 2022). It is also consistent with research suggesting that designing learning opportunities requires teachers to make prospective judgments about learners and their likely responses (Brunner et al., 2021; Nathan & Koedinger, 2000).

AI-supported interaction may have provided an additional context for this process by offering an interactive environment in which PSTs could externalize, examine, and revise their thinking. Rather than functioning simply as a source of ready-made tasks, AI-generated suggestions became objects that PSTs could accept, question, or modify during the design process. In this sense, AI-supported iterative design may provide additional occasions for PSTs to connect professional knowledge with concrete design decisions. This interpretation is consistent with research emphasizing the potential of AI-supported learning environments to provide adaptive scaffolding and support professional learning (Faber et al., 2024; Yan et al., 2025), as well as the importance of teacher agency and critical engagement in human–AI collaboration (Kim, 2026; Uygun et al., 2025). However, the present design does not allow the observed pre–post changes to be attributed specifically to these AI-supported interactions. The changes should instead be interpreted in the context of the full module, which also involved instruction, repeated task design practice, reflection, and feedback.

### 6.2. Configurational Pathways to High Diagnostic Thinking

The fsQCA results identified two configurations associated with high post-intervention diagnostic thinking, suggesting that this outcome may be associated with more than one combination of conditions. However, the two configurations showed different levels of stability across the robustness analyses; accordingly, H1 is treated as the comparatively stable configuration, whereas H2 is treated as a tentative, sensitivity-dependent configuration. These findings complement the paired-samples analysis by showing that high post-intervention diagnostic thinking was associated with different combinations of learner characteristics and AI interaction. Given the pilot nature of the study and the relatively small sample, these configurational findings should be interpreted as exploratory and require validation in larger samples. In particular, high AI acceptance was present in both successful configurations, whereas initial diagnostic thinking and prompting knowledge density varied across pathways. Together, these findings highlight the configurational nature of high post-intervention diagnostic thinking, rather than suggesting that any single learner characteristic alone explains the outcome.

High AI acceptance appeared in both configurations. This finding is consistent with research suggesting that users’ perceptions of the usefulness and value of AI influence their willingness to engage with the technology (Nikolopoulou et al., 2020; Tan et al., 2025; Wang et al., 2025). However, the present findings provide a more differentiated interpretation of this relationship. AI acceptance alone was not sufficient for high diagnostic thinking; rather, it appeared to operate in combination with different levels of initial diagnostic thinking and prompting knowledge density. Thus, positive attitudes toward AI may support sustained engagement, but how learners use AI and what they gain from that engagement may depend on their existing competence and interactional behavior.

A second important finding is that a high initial level of diagnostic thinking was not necessary for achieving a high final level. The stable H1 configuration suggests that PSTs with relatively low initial diagnostic thinking could nevertheless achieve high outcomes when combined with high AI acceptance and high prompting knowledge density. This finding is broadly consistent with research on adaptive scaffolding, which suggests that learners with different levels of prior knowledge may benefit from different forms of support (Chernikova et al., 2020). At the same time, the present study extends this perspective by showing that the differences may not only concern the amount of support learners need but also the way they engage with AI during professional learning.

The exploratory contrast between H1 and H2 further suggests that prompting knowledge density may be associated with different patterns of cognitive engagement. PSTs with lower initial diagnostic thinking tended to produce more knowledge-dense prompts, potentially using AI to obtain broader support concerning mathematical content, student thinking, and task difficulty. By contrast, those with higher initial diagnostic thinking in exploratory H2 configuration could engage in more selective and targeted interactions, relying more extensively on their existing professional knowledge to evaluate and refine AI-generated suggestions. These contrasting patterns raise the possibility that the role of AI support may differ according to PSTs’ existing diagnostic competence, ranging from broader knowledge-oriented support to more targeted verification and refinement.

### 6.3. Differentiated Human–AI Interaction Across Pathways

Whereas the configurational analysis identified combinations of conditions associated with high final diagnostic thinking, the qualitative analysis provided a process-level view of how PSTs engaged with, evaluated, and revised AI-generated suggestions during task design. The findings suggest that AI was used not only to generate or modify task content, but also to check emerging judgements, evaluate the appropriateness of AI-generated outputs, and support subsequent revision decisions. Within this broader pattern, cases in H1 and the tentative H2 configuration showed some exploratory differences in how these processes unfolded. Participants in the H1 configuration, who entered the module with relatively lower initial diagnostic thinking, more frequently used AI to explore task difficulty, curriculum alignment, and possible student understanding. Their prompts often sought broader forms of knowledge and guidance, suggesting that AI functioned as a source of external support for constructing and organizing professional knowledge. In contrast, the H2 cases, who began with higher levels of diagnostic thinking, engaged in more selective interactions, focusing on particular aspects of task presentation and representation. This exploratory contrast suggests that AI may be used in different ways during task design, ranging from checking relatively tentative judgements to refining specific aspects of an emerging task.

The findings therefore provide a more nuanced account of AI-supported professional learning. Rather than simply showing that some participants use AI more frequently or more effectively than others, the results suggest that participants with different initial levels of diagnostic thinking may use AI for different purposes. Participants with lower initial diagnostic thinking may require broader knowledge-oriented support, whereas those with stronger initial diagnostic thinking may use AI more selectively to verify, refine, or challenge specific aspects of their own reasoning. This interpretation is consistent with research on differentiated scaffolding and the role of prior knowledge in learning from instructional support (Chernikova et al., 2020).

The qualitative findings also highlight the importance of maintaining professional agency during AI-supported learning. The H2 cases, in particular, provide examples of PSTs identifying inaccuracies or inappropriate levels of difficulty in AI-generated content and responding with more precise instructions. Such interactions suggest that effective AI collaboration involves not only obtaining information from AI but also evaluating the relevance and validity of its suggestions. This point is important because AI-generated outputs may contain inaccuracies or may not adequately reflect curricular and pedagogical requirements (Ergene & Ergene, 2025). Accordingly, the development of PSTs’ capacity to critically evaluate AI outputs while maintaining professional judgement may be an important component of AI-supported professional learning. At the same time, diagnostic thinking primarily concerns how teachers attend to, interpret, and judge students’ possible mathematical thinking; it does not in itself provide a comprehensive framework for evaluating the broader didactical suitability of AI-generated tasks. This distinction points to an important direction for extending research on teachers’ professional judgement in AI-supported task design.

In sum, the findings suggest that AI-supported mathematical task design may support the development of PSTs’ understanding of students’ mathematical thinking through different interactional pathways. The H1 cases with lower initial diagnostic competence suggest a more exploratory use of AI for checking and developing emerging judgements about students and task appropriateness, whereas the tentative H2 cases with higher initial competence illustrate more targeted uses of AI for evaluating and refining specific task features. The findings therefore point to the importance of considering how AI use interacts with learners’ existing professional competence, rather than assuming that a single form of human–AI interaction is optimal across participants.

## 7. Conclusions and Implications

This study examined how pre-service mathematics teachers’ diagnostic thinking changed over a six-week program of AI-supported mathematical task design. Participants showed significantly higher post-test scores in overall diagnostic thinking and in the dimensions of perception, interpretation, and judgement than at pre-test. The configurational analysis identified one comparatively stable configuration associated with high post-intervention diagnostic thinking, characterized by lower initial diagnostic thinking, high AI acceptance, and high prompting knowledge density. A second configuration, involving higher initial diagnostic thinking, high AI acceptance, and low prompting knowledge density, emerged in the primary analysis but was sensitivity-dependent and is therefore treated as tentative. Qualitative comparisons suggested that participants in H1 tended to use more knowledge-dense prompts to seek broader support, whereas the H2 cases appeared to use AI more selectively for verification and refinement. Taken together, these findings suggest that AI-supported professional learning may involve heterogeneous forms of interaction rather than a single uniform pattern.

Theoretically, this study contributes to research on teacher learning and human–AI collaboration in three ways. First, it extends the empirical scope of research on diagnostic thinking by showing that mathematical task design can provide a context for prospective reasoning about students’ possible mathematical thinking, rather than focusing exclusively on the interpretation of already-produced student responses. Second, the configurational findings highlight the value of examining combinations of learner characteristics and interactional behaviors rather than focusing solely on the independent effects of individual variables. In particular, the comparatively stable H1 configuration indicates that high post-intervention diagnostic thinking was associated with the combination of lower initial diagnostic thinking, high AI acceptance, and high prompting knowledge density. Third, the study contributes to understanding human–AI collaboration as a differentiated process. The findings suggest that AI may function as a source of broader knowledge-oriented scaffolding for learners with lower initial diagnostic thinking, while serving as a tool for targeted verification and critical refinement for learners with higher initial diagnostic thinking. This perspective offers a more nuanced account of how AI may serve different functions in professional learning depending on PSTs’ existing diagnostic competence and patterns of interaction.

Practically, the findings suggest that AI integration in teacher education should move beyond teaching PSTs how to use AI tools or generate instructional materials. Teacher education programs should create structured activities in which PSTs must anticipate student thinking, formulate pedagogically meaningful prompts, evaluate AI-generated suggestions, and revise their decisions through reflection. The findings also suggest that AI-supported learning activities should be sensitive to differences in learners’ initial diagnostic thinking. The exploratory differences observed across the selected cases raise an important question for future research: whether PSTs with different levels of initial diagnostic thinking may benefit from different forms of AI support. Larger-scale studies are needed to examine whether broader knowledge-oriented support and more selective verification and refinement are differentially useful for PSTs with different levels of diagnostic competence. Across participants, however, developing the capacity to critically evaluate AI outputs and maintain professional agency remains important.

Several limitations should nevertheless be acknowledged. First, the relatively small sample was drawn from a specific teacher-education context; thus, the configurational findings should be interpreted as exploratory rather than generalizable. The H2 configuration was also sensitive to alternative calibration and PRI specifications and should therefore be treated cautiously. Future studies with larger, multi-institutional samples are needed to examine whether similar patterns emerge across contexts. Second, some limitations concern measurement and research design. Although Forms A and B were designed to be similar in mathematical content domains, task structure, and diagnostic prompts, the small AB/BA group sizes limit the extent to which the sequence comparison can establish full equivalence between forms. Moreover, the single-group pre–post design limits attribution of observed changes specifically to AI-supported task design, as repeated task design practice, reflection, feedback, and other learning experiences may also have contributed to participants’ development. Third, although the Judgement scoring procedure was based on empirically informed reference success rates and detailed scoring criteria, the exact item-level reference rates the exact item-level reference rates could not be publicly reported. Future studies should provide more detailed benchmark information to further enhance the transparency of Judgement scoring. In addition, reliance on a single generative AI platform (DeepSeek V3) means that some interaction patterns may reflect model- or interface-specific features, warranting replication across different AI models and platforms. The study also provided limited direct evidence of the moment-to-moment cognitive processes underlying perception, interpretation, and judgement; future research could combine interaction-log analysis with eye-tracking, think-aloud protocols, or stimulated-recall interviews. Beyond these methodological extensions, future studies could also draw on frameworks such as the Didactic Suitability Criteria to examine how PSTs evaluate and refine the broader didactical suitability of AI-generated tasks, including how they recognize and revise their own potentially inaccurate mathematical or pedagogical judgements (Font et al., 2024).

## Figures and Tables

**Table 1:** The operational definitions and coding scheme of prompts.

Score	Type	Description	Example
4	(a) Integrated PCK-CK Focus	The prompt explicitly connects mathematical content with student cognition.	“Modify the component regarding linear equations to reduce the difficulty and adapt it to the level of seventh-grade students.”
3	(b) Isolated Knowledge Focus	The prompt focuses on targeting either PCK or CK exclusively.	“The trajectory equation you provided contains a mathematical error.”
2	(c) Surface Feature Focus	The prompt focuses on tasks’ surface features, such as the contextual setting, language fluency, or formatting.	“Revise the phrasing of the question to be more rigorous.”
1	(d) General Improvement Focus	The prompt asks the AI to revise the task without specifying a particular mathematical or pedagogical requirement.	“Please further revise the problem.”

**Table 2:** Data calibration.

Type	Variables	Anchors			Descriptive Statistics			
		Full Membership (0.95)	Crossover Point (0.5)	Full Non-Membership (0.05)	Mean Value	Standard Value	Maximum Value	Minimum Value
Conditional variables	IDT ^1^	12	8	2	7.95	3.32	13	2
	ACC ^2^	4.40	3.66	3.34	3.77	0.44	4.48	2.94
	PKD ^3^	0.91	0.71	0.43	0.69	0.13	0.94	0.39
Outcome variable	FDT ^4^	15	13	9	12.67	2.22	16	7

^1^ IDT initial diagnostic thinking; ^2^ ACC acceptance towards AI; ^3^ PKD prompting knowledge density in chatlogs; ^4^ FDT final level of diagnostic thinking.

**Table 3:** Paired-samples *t*-test results for diagnostic thinking dimensions (N = 21).

	Pre-Test M (SD)	Post-Test M (SD)	Mean Diff ^1^	SD of Diff ^2^	95% CI ^3^		t ^4^	df ^5^	*p* ^6^	Cohen’s d ^7^
					Lower	Upper				
Perception	3.429 (1.805)	5.143 (0.910)	1.714	1.821	0.886	2.543	4.315	20	<0.001	0.94
Interpretation	2.762 (1.729)	4.476 (1.209)	1.714	1.586	0.993	2.436	4.954	20	<0.001	1.08
Judgement	1.762 (1.136)	3.048 (0.740)	1.286	1.384	0.656	1.916	4.258	20	<0.001	0.93
Overall DT ^8^	7.952 (3.324)	12.667 (2.221)	4.714	3.258	3.231	6.197	6.631	20	<0.001	1.45

^1^ Reported values include mean differences, ^2^ standard deviations (SD) of differences, ^3^ 95% confidence intervals, ^4^ t statistics, ^5^ degrees of freedom (df), ^6^ two-tailed significance levels, ^7^ effect size, ^8^ DT means diagnostic thinking.

**Table 4:** Necessary condition analysis results.

Configurational Conditions	FDT		~FDT *	
	Consistency	Coverage	Consistency	Coverage
IDT	0.659872	0.666906	0.592734	0.515808
~IDT	0.520915	0.597661	0.617229	0.609761
ACC	0.670241	0.735629	0.480445	0.454041
~ACC	0.50257	0.529061	0.720255	0.652859
PKD	0.710209	0.742656	0.501955	0.451951
~PKD	0.475895	0.526006	0.714183	0.679694

* ‘~’ means the condition does not exist. IDT initial diagnostic thinking; ACC acceptance towards AI; PKD prompting knowledge density in chatlogs; FDT final level of diagnostic thinking.

**Table 5:** Configurations linked to high/low final diagnostic thinking.

Condition Variable	High FDT		Low FDT
	H1	H2	L1
IDT	⊗ ^1^	√ ^2^	⊗
ACC	√	√	⊗
PKD	√	⊗	⊗
Raw coverage	0.303	0.305	0.400
Unique coverage	0.163	0.165	0.400
Consistency	0.881	0.825	0.931
Overall Solution coverage	0.468		0.400
Overall Solution consistency	0.853		0.931

^1^ ⊗ means this condition does not exist; ^2^ √ means that this condition exists. IDT initial diagnostic thinking; ACC acceptance towards AI; PKD prompting knowledge density in chatlogs; FDT final level of diagnostic thinking.

**Table 6:** Thematic Classification and Frequency Distribution of PSTs’ AI Prompts.

Type	Code	Theme	Frequency
(A) Integrated PCK-CK Focus	A#1	Align content with student ability	25
	A#2	Align task context with student understanding	50
(B) Isolated Knowledge Focus	B#1	Adjust the knowledge points tested	31
	B#2	Adjust the design logic of the question chain	43
	B#3	Adjust task difficulty to grade level	69
	B#4	Adjust task in line with curriculum standards	94
(C) Surface Feature Focus	C#1	Ensure task is readable and accessible	41
	C#2	Revise task format or illustration	38
(D) General Improvement Focus	D#1	Make general revisions to improve clarity	37
	D#2	Require AI to self-check or reflect	10

## Data Availability

The first author can provide access to the datasets generated during the current study upon receiving a reasonable request.

## Supplementary Materials

The following supporting information can be downloaded at: https://www.mdpi.com/article/10.3390/jintelligence14090227/s1, Table S1: Truth table for High Final Diagnostic Thinking; Table S2: Truth table for Low Final Diagnostic Thinking; Table S3: Sensitivity Analysis of fsQCA Solutions.

## Abbreviations

The following abbreviations are used in this manuscript:

LLMs	Large language models
MKT	Mathematical Knowledge for Teaching
PCK	Pedagogical Content Knowledge
CK	Content Knowledge
PKD	prompting knowledge density in chatlogs
fsQCA	fuzzy-set Qualitative Comparative Analysis
ACC	acceptance towards AI
IDT	initial diagnostic thinking
FDT	final level of diagnostic thinking

## Appendix A

Please carefully read the problem statement above. Reflect on the mathematical knowledge, reasoning processes, and potential student responses involved in solving this problem, and answer the following questions:
(I) What prerequisite mathematical knowledge and key mathematical features should students recognize to solve this problem?
(II) What errors or difficulties might students encounter when solving this problem, and what mathematical reasoning might underlie them?
(III) What proportion of the target students do you expect to solve this problem successfully, and what is the basis for your estimate?

Please carefully read the problem statement above. Reflect on the mathematical knowledge, reasoning processes, and potential student responses involved in solving this problem, and answer the following questions:
(I) What prerequisite mathematical knowledge and key mathematical features should students recognize to solve this problem?
(II) What errors or difficulties might students encounter when solving this problem, and what mathematical reasoning might underlie them?
(III) What proportion of the target students do you expect to solve this problem successfully, and what is the basis for your estimate?

## Appendix B

Please carefully read the problem statement above. Reflect on the mathematical knowledge, reasoning processes, and potential student responses involved in solving this problem, and answer the following questions:
(I) What prerequisite mathematical knowledge and key mathematical features should students recognize to solve this problem?
(II) What errors or difficulties might students encounter when solving this problem, and what mathematical reasoning might underlie them?
(III) What proportion of the target students do you expect to solve this problem successfully, and what is the basis for your estimate?

Please carefully read the problem statement above. Reflect on the mathematical knowledge, reasoning processes, and potential student responses involved in solving this problem, and answer the following questions:
(I) What prerequisite mathematical knowledge and key mathematical features should students recognize to solve this problem?
(II) What errors or difficulties might students encounter when solving this problem, and what mathematical reasoning might underlie them?
(III) What proportion of the target students do you expect to solve this problem successfully, and what is the basis for your estimate?

## Appendix C

**Table A1:** The operational definitions and coding scheme of diagnostic thinking.

Dimension	Score	Description	Example
Perception	0	Fails to provide the correct answer or identifies inaccurate or biased knowledge points.	This problem requires knowledge of solid geometry.
	1	Mentions knowledge points in a generic way without linking them to the specific problem context.	It examines the content about the positional relationship between a straight line and a plane.
	2	Identifies one specific knowledge point required for solving the problem	The students need to master how to prove a straight line is parallel to a plane.
	3	Accurately identifies two or more specific knowledge points for solving the problem.	Students need to master how to prove a straight line is parallel to a plane and apply the property that lines and planes are perpendicular.
Interpretation	0	The analysis is irrelevant to the specific difficulties of the task.	The students might make mistake if they did not read the question carefully.
	1	Point out part of the difficulties of the task but not accurate.	It is difficult for students to build connections between geometry and algebra.
	2	Explains the task difficulty by linking it to general knowledge but fails to identify the core difficulty.	It is difficult for students to build space rectangular coordinate system.
	3	Describe typical student errors by integrating specific problem content and features.	The students mistakenly assume the lateral edge of the triangular prism is perpendicular to the base and fail to prove the rationality of space rectangular coordinate system.
Judgement	0	The absolute difference from the reference success rate is greater than 20 percentage points.	
	1	The absolute difference from the reference success rate is greater than 10 and no more than 20 percentage points.	
	2	The absolute difference from the reference success rate is 10 percentage points or less.	

## Appendix D

**Table A2:** Part of a survey assessing PSTs acceptance of AI.

Sub-Dimension	Examples Item in the Scale
Performance Expectancy	I believe that using AI Chatbots is beneficial in mathematics task design.
Effort Expectancy	Learning how to use AI Chatbots is easy for me.
Social Influence	I am influenced by my friends to use AI Chatbots for mathematics task design.
Facilitating Conditions	I possess the knowledge and skills required to use AI Chatbots for mathematics task design.
Habit	I have developed a habit of using AI Chatbots for learning and teacher practice regularly.
Hedonic Motivation	I think using AI Chatbots for learning mathematics and teacher practice for studying is interesting
Behavioral Intention	I will recommend AI Chatbots to my friends for mathematics task design.
Usage Behavior	I use AI Chatbots to create mathematics questions.
