# Supplementary material for: Exploring Pre-Service Mathematics Teachers’ Diagnostic Thinking During AI-Supported Task Design: A Configurational Mixed-Methods Pilot Study
Source: J Intell. 2026 Sep 20;14(9):227. doi: 10.3390/jintelligence14090227 (PMC13608781; doi:10.3390/jintelligence14090227)
Supplement: Supplementary file 1 [file jintelligence-14-00227-s001.zip › jintelligence-4499401-supplementary.pdf]

Supplementary Table S1. Truth table for High Final Diagnostic Thinking

IDT	PKD	ACC	Cases (n)	Raw consistency	PRI consistency	FDT
0	1	1	2	0.881	0.771	1
1	0	1	3	0.825	0.718	1
0	1	0	2	0.816	0.643	0
1	1	1	4	0.811	0.635	0
1	1	0	3	0.816	0.627	0
0	0	1	2	0.629	0.380	0
1	0	0	3	0.592	0.238	0
0	0	0	2	0.479	0.117	0

Supplementary Table S2. Truth table for Low Final Diagnostic Thinking

IDT	PKD	ACC	Cases (n)	Raw consistency	PRI consistency	~FDT
0	0	0	2	0.931	0.883	1
1	0	0	3	0.793	0.614	0
0	1	0	2	0.723	0.537	0
1	0	1	3	0.660	0.313	0
1	1	0	3	0.554	0.282	0
0	1	1	2	0.601	0.229	0
0	0	1	2	0.601	0.227	0
1	1	1	4	0.532	0.097	0

Supplementary Table S3. Sensitivity Analysis of fsQCA Solutions

Test	Alternative setting	Result
Calibration	90/50/10	H1, L1 retained; H2 disappeared
Frequency	cutoff=2	H1, H2, L1 retained
Consistency	0.82	H1, H2, L1 retained
PRI	0.75	H1, L1 retained; H2 disappeared
